# The impact of endometrial thickness (EMT) on the day of human chorionic gonadotropin (hCG) administration on pregnancy outcomes: a 5-year retrospective cohort analysis in Malaysia

**DOI:** 10.1186/s40738-018-0050-8

**Published:** 2018-08-11

**Authors:** Joe Mee Chan, Alvin Isaac Sukumar, Magendra Ramalingam, Surinder Singh Ranbir Singh, Mohamad Farouk Abdullah

**Affiliations:** 1 Hospital Tengku Ampuan Rahimah, Jalan Langat, 41200 Klang, Selangor Malaysia; 2 Tropicana Medical Center, Jalan Teknologi, PJU 5 Kota Damansara, 47810 Petaling Jaya, Malaysia

**Keywords:** In vitro fertilization(IVF), Embryo transfer(ET), Endometrial thickness(EMT), Thin endometrium, Endometrial receptivity, Ultrasound, Pregnancy

## Abstract

**Background:**

Chances of pregnancy in relation to endometrial thickness (EMT) remain elusive albeit some literatures suggest poorer pregnancy outcomes below the threshold of 6-7 mm, notwithstanding others perceive detrimental effect at thicker EMT. We aim to examine the implication of EMT on pregnancy outcomes using a cut-off of 8 mm and further explore for any effect of ‘thick’ EMT in our patient population.

**Methods:**

This was a retrospective cohort study performed for 162 women to assess the associations between EMT on the human chorionic ganadotropin (hCG) trigger day and pregnancy outcomes in infertile patients undergoing in vitro fertilization (IVF)/intracytoplasmic sperm injection (ICSI) and autologous fresh embryo transfer (ET) in controlled ovarian stimulation (COS) cycles under an assisted reproductive technology (ART) shared-care programme between public and private institutions from January 2012 through December 2016.

The associations between pregnancy outcomes [Total Pregnancy Rate (TPR), Biochemical Pregnancy Rate (BPR), Clinical Pregnancy Rate (CPR), Ongoing Pregnancy Rate (OPR)/ Live Birth Delivery Rate (LBDR), Miscarriage Rate (MR) and Implantation Rate (IR)] and EMT (< 8 or ≥ 8 mm) on the hCG trigger day were evaluated. Besides, the associations between pregnancies outcomes with EMT ≥ 14 mm and ≥ 8 to < 14 mm were further assessed.

**Results:**

We found that the ≥8 mm group had a higher TPR (55.4% vs 21.4%; *p* = 0.015) and CPR (52.0% vs 21.4%; *p* = 0.029). However, the BPR, MR, OPR/ LBDR and IR were not associated with the EMT (*p* > 0.05). All pregnancy outcomes were comparable for ≥14 mm and ≥ 8 to < 14 mm subgroups.

**Conclusions:**

Our findings suggest that EMT < 8 mm on hCG trigger day could adversely affect TPR and CPR in infertile patients undergoing IVF/ICSI-ET. Besides, we also disprove the notion of reduced chances of pregnancy with EMT ≥ 14 mm. The findings are based on completed cycles which each has demonstrated a triple-line endometrial pattern on the hCG trigger day with fresh autologous ET consisted of high-quality morphological gradings. However, our findings are still preliminary to suggest decision for ET transfer, cycle cancellation or adjunctive therapies. Further studies with larger sample size from this geographical region are required to verify the findings.

## Background

Successful in vitro fertilization (IVF)/ intracytoplasmic sperm injection (ICSI) cycle outcome depends on intricate interplay between endometrial receptivity and embryo quality. The endometrial receptivity for early embryo implantation during the “implantation window” is characterized by certain morphological and functional alternations. One of the observed alterations includes a dynamic increase in the proliferation and thickness of the endometrium approaching periovulatory phase.

Endometrial thickness (EMT) measurement is an easy ultrasonographic technique to perform. Besides, it has minimal intraobserver and interobserver variability, hence a reliable tool for assessing endometria in patients undergoing controlled ovarian stimulation (COS) [1].

Albeit there are many literatures which look into the relationship between endometrial thickness and receptivity [2–4], and accepting that EMT assessment in the midsagittal plane via transvaginal ultrasound (TVU) is a standard practice in assisted reproductive technology (ART) centres worldwide, the demonstration of clinical significance of EMT as an independent determinant of IVF outcomes till date remains controversial. There is currently no agreement regarding the EMT necessary for successful conception.

Several study groups based on autologous fresh embryo transfer (ET) cycles had reported improved pregnancy rates with increasing EMT [5–8], but others did not establish a significant relationship between EMT and the chance to conceive [8, 9]. Numerous studies had taken a closer look at variable cut-off values between 6 and 10 mm on the day of human chorionic gonadotropin (hCG) trigger, below which, pregnancy rates (PRs) were shown to be significantly lower. While most had previously ascribed to a cut-off value of 7 mm, pooled-data from recent systemic review and meta-analysis seemed to show probable greater clinical significance with an EMT cut-off between 8 and 10 mm [8].

On the upper limit, EMT of 14 mm was found in approximately 5–10% of patients who had reached the ET stage [10]. Applebaum et al. in their earlier work had assigned a low score of 1 to an EMT > 14 mm in a uterine scoring system for reproduction developed by them looking at the uterine biophysical profile that would favour conception [11]. A threshold of 14 mm had been regarded as ‘thick’ endometrium and subjected for assessment in subsequent studies. Weissman et al. [10] reported that implantation and pregnancy rates were significantly reduced, and miscarriage rates might be increased when the EMT measured > 14 mm on the day of hCG trigger, while Kupesic et al. reported no pregnancies with an EMT > 15 mm on the day of ET [12]. On the other hand, Dietterich et al. [13], reported no difference in the clinical pregnancy rate (CPR) when the EMT was 14 mm, while Rodolfo et al. reported successful pregnancies in the setting of exaggerated EMT of 16 mm and 20 mm in two of their IVF patients [14].

Till date, there is lacking of consensus on the discerning threshold or cut-off values of EMT vital for conception and pregnancy, thus render decision-making on continuing IVF treatment or resorting to elective frozen-thawed embryo transfer (FET) a contentious subject, whilst indications and justifications remain equivocal for any proclaimed therapeutic intervention. There is generally a paucity of literature to address these basic concerns especially within the Southeast Asian patient populations undergoing COS for IVF/ICSI-ET.

This present study was set to investigate the EMT threshold values on the day of hCG trigger that would favour conception in the fresh autologous ET cycles of patients undergoing COS and IVF/ICSI for infertility. We hypothesized that EMT ≥8 mm was associated with better pregnancy outcomes. Subgroup analyses were further carried out to examine pregnancy outcomes with each 1 mm EMT increment and to evaluate the effect of an arbitrary ‘thick’ endometrium on pregnancy outcomes. This study was to the best of our knowledge the first within the Southeast Asian region to ascertain the cut-off value of 8 mm EMT for any statistically significant distinguishing effects on pregnancy outcomes.

## Methods

This retrospective cohort study included 162 consecutive autologous fresh IVF/ICSI-ET cycles performed between January 2012 and December 2016 under the shared-care programme between Tengku Ampuan Rahimah Hospital Klang and Tropicana Medical Centre, Kota Damansara in the Selangor state in Malaysia. Under this Ministry of Health’s ART programme, eligibility was applicable to any married couple (with either spouse possessing a Malaysian nationality) who had no biological living child and whose female spouse’s age was less than 43.

Patients were excluded from the study if they were found to have abnormal uterine cavity as demonstrated by hysterosalpingography, saline sonohysterogram or hysteroscopy, presence of a known endometrial polyp, uterine anomaly, uterine fibroid, adenomyosis, and/ or hydrosalpinx. Among other risk factors for defective endometrial receptivity, given as previous pelvic inflammatory disease (PID), previous ectopic pregnancy, endometriosis, previous uterine instrumentation [eg. history of dilation and curettage (D&C)] or surgery (eg. history of myomectomy breaching endometrial cavity), and previous endometritis (eg. with multiple prior miscarriages or uterine infection caused by miscarriage) were not excluded from the study.

EMT on the hCG trigger day was not used as a criterion for cancellation of oocyte retrieval or embryo transfer in our setting. However, subjects were excluded from the analysis in the absence of TVU demonstration of triple-line endometrial pattern on the hCG trigger day. Cycles using preimplantation genetic screening (PGS), preimplantation genetic diagnosis (PGD), donor gametes, or cryopreserved-thawed embryos were also excluded. Only patients transferred with high-quality cleavage-stage embryos or blastocyst were included in this study. For patients who underwent multiple treatment cycles during the period of the study, only the first cycles were included.

The study was consisted of female patients whose either or both of the couple were confirmed the diagnoses of female and/or male factor(s), or unexplained infertility. COS were carried out using either the gonadotropin-releasing hormone (GnRH) antagonist (*n* = 78; 48.1%) or agonist (*n* = 84; 51.9%) protocol. The choice of COS protocol and the starting dose of gonadotropin were based on clinicians’ careful consideration of the individual pretreatment characteristics, personal infertility history and/ or any identified cause(s) of infertility. Subcutaneous hCG 10,000 IU was administered to trigger the final maturation of oocytes when the follicular growth was deemed optimal for the patient.

EMT was defined as the maximal distance between the echogenic interfaces of the myometrium and the endometrium [8]. All examinations were performed using a two-dimensional TVU (Capasee II Ultrasound, SSA-220A with PVG-601 V 7.0 MHz transvaginal probe, Toshiba, Tokyo, Japan) for EMT at the midsagittal plane on the day of ovulation trigger.

Oocyte retrieval was done under general anaesthesia by TVU-guided aspiration 35–37 h after ovulation triggering. IVF/ICSI-ET procedures were carried out as per indicated. Embryos and blastocysts derived from IVF or ICSI were graded as good, fair or poor according to the Society for Assisted Reproductive Technology (SART) ‘s standardization of grading embryo morphology [15]. A maximum of 2–3 cleavage-stage embryos were transferred after 2 or 3 days of growth in culture media or a single blastocyst was transferred on day-5 or − 6 of culture. Should there be > 3 high-quality embryos on Day 2/3, the couple would be given the option of extended culture to Day 5/6 to allow for blastocyst transfer. In order for couple to make an informed choice, detailed counselling would be given which included information about the risk of no transfer as a result of embryos not progressing to blastocyst stage. There were no set rules about timing of cryopreservation for surplus embryos as each preferred timing varied and was thus individualised. Only ETs consisted of ascertained high-quality morphological gradings (graded as good and/ or fair) were included in this study. All patients received their luteal support beginning on the day of oocyte retrieval (OR), consisting of 90 mg of vaginal progesterone gel (Crinone 8%) twice-daily administered until 8 weeks of gestation followed by 200 mg of vaginal progesterone capsules (Utrogestan) thrice-daily until 12 weeks of gestation. Besides, oral estradiol valerate tablets (Progynova) 2 mg thrice-daily were supplemented for 4 weeks starting from the day of OR.

Serum hCG levels were assessed two weeks after oocyte retrieval and ultrasound scan confirmation of all pregnancies was performed on all patients between five to six weeks estimated gestational age based on ET day. Total Pregnancy Rate (TPR) in our study was defined as overall pregnancy rates (PRs) comprising of those arising from biochemical or clinical pregnancies. Miscarriage Rate (MR) was defined as rates of spontaneous miscarriages (including ectopic pregnancies). The definitions for biochemical pregnancy, clinical pregnancy, spontaneous miscarriage, CPR, Live Birth Delivery Rate (LBDR), and Implantation Rate (IR) were according to the ICMART and the WHO revised glossary of ART terminology, 2009 [16]. Ongoing pregnancy was defined when the pregnancy had completed ≥20 weeks of gestation [17]. The specified denominator for TPR, Biochemical Pregnancy Rate (BPR), CPR, Ongoing Pregnancy Rate (OPR) / LBDR for our study was per ET cycle. Poor Ovarian Response (POR) was defined as ≤3 oocytes retrieved with a conventional stimulation protocol [18].

Based on literature review and the agreed observation of our center local database, we decided to use the cut-off value of 8 mm, which corresponded to the 10th centile value of EMT in our patient population, with which, all our patients were divided into two main groups, ie. Group A (< 8 mm) or Group B (≥8 mm) for the analyses of cycle outcomes. For the subgroup analyses, EMT cut-off value of 14 mm which corresponded to the upper 90th to 95th centile was used to evaluate pregnancy outcomes between women with EMT ≥ 14 mm and EMT ≥8 to < 14 mm.

Patient’s characteristics were compared between the two main EMT groups for any significant association or difference. Outcomes of COS, IVF/ICSI and pregnancy outcomes were first compared between the 2 main EMT groups and subsequent comparisons were done for pregnancy outcomes between the two subgroups.

The Malaysian Research and Ethics Committee had approved the study (NMRR ID 16–2868-33,076).

### Statistical analysis

Continuous variables were presented as mean ± standard deviation (SD) and the comparisons were tested by student’s t-test. Comparisons of proportions were made by the Chi-square test and Fisher’s exact test. Statistical significance was defined as *p* < 0.05. All analyses were performed by using SPSS statistical package for Windows, version 11.0 (SPSS Inc., Chicago, IL).

## Results

All women in our study were aged between 26 and 43 years of age, and had body mass indices (BMI) between 16.0 and 42.0 kg/m^2^. The EMT of our patient population ranged from 5.2 to 19 mm on the day of ovulation trigger. The majority of our patients had an EMT between ≥8 to < 14 mm (*n* = 134/162, 82.7%), with EMT of 8 mm falling on the 10th centile whilst EMT of 14 mm falling between the 90th to 95th centile.

Our patients’ characteristics were comparable between the group with EMT < 8 mm and ≥ 8 mm (Table 1). We did not find any statistically significant difference between the < 8 mm and ≥ 8 mm groups with respect to the number of oocytes retrieved, mature oocytes retrieved and fertilized oocytes (Table 2).

**Table 1:** Patients’ demographic and pretreatment characteristics

	< 8 mm	≥8 mm	*p*
No. of ET cycles	*n* = 14(8.6%)	*n* = 148 (91.4%)	Total *n* = 162
Ethnicity:			
Malay	4/14 (28.6%)	64/148 (43.2%)	NS
Chinese	4/14 (28.6%)	33/148 (22.3%)	NS
Indian	6/14 (42.8%)	50/148 (33.8%)	NS
Others	0/14 (0%)	1/148 (0.7%)	NS
Age of patients (years)	32.9 ± 4.2	33.9 ± 3.6	NS
Infertility duration (years)	5.4 ± 2.8	6.1 ± 3.4	NS
BMI (kg/m^2^)	24.3 ± 4.7	25.1 ± 4.7	NS
Basal FSH (IU/L)	6.1 ± 1.5	7.2 ± 3.1	NS
Basal LH (IU/L)	4.9 ± 2.3	5.5 ± 2.8	NS
Basal E2 (pmol/L)	46.9 ± 30.9	91.7 ± 64.9	NS
Causes of Infertility:			
Tubal/ Peritoneal	8 (57.1%)	51 (34.5%)	NS
Ovulatory dysfunction	5 (35.7%)	50 (33.8%)	NS
Male factor	6 (42.9%)	56 (37.8%)	NS
Unexplained	1 (7.1%)	29 (19.6%)	NS

Values were expressed in means ± SD; NS = not significant

*BMI* Body Mass Index, *FSH* Follicle-stimulating hormone, *LH* Luteinizing Hormone, *E2* Estradiol

**Table 2:** Comparisons of treatment measures, outcomes of COS, IVF/ICSI and PRs

	< 8 mm	≥8 mm	*p*
No. of ET cycles	14	148	Total *n* = 162
No. of GnRH antagonist cycles	50.0% (7/14)	48.0% (71/148)	NS
Days of Gn	10.1 ± 2.0	10.4 ± 1.9	NS
Total dose of Gn (IU)	2756.7 ± 1148.0	2850.1 ± 1062.1	NS
E2 on hCG day (pmol/L)	8608.9 ± 5132.1	6762.4 ± 5126.7	NS
Progesterone on hCG day (nmol/L)	2.1 ± 1.1	2.7 ± 1.8	NS
EMT on hCG day (mm)	6.9 ± 0.8	10.8 ± 2.1	0.000^*^
No. of oocytes retrieved	12.7 ± 6.0	13.7 ± 7.9	NS
No. of mature oocytes	9.6 ± 4.9	11.2 ± 7.1	NS
No. of fertilized oocytes	6.1 ± 3.7	7.3 ± 4.9	NS
No. of embryos transferred	2.1 ± 0.5	2.2 ± 0.6	NS
- No. of good embryos	1.7 ± 0.6	1.4 ± 0.9	NS
- No. of fair embryos	0.4 ± 0.8	0.8 ± 0.8	NS
No. of ET cycles using blastocyst	7.1% (1/14)	11.5% (17/148)	NS
No. of embryos cryopreserved	1.4 ± 1.7	2.2 ± 2.7	NS
No. of cycles with POR	0% (0/14)	5.4% (8/148)	NS
No. of cycles with ICSI	64.3% (9/14)	62.8% (93/148)	NS
TPR per ET cycle (%)	21.4% (3/14)	55.4% (82/148)	0.015^*^
BPR per ET cycle (%)	0% (0/14)	3.4% (5/148)	NS
CPR per ET cycle (%)	21.4% (3/14)	52.0% (77/148)	0.029^*^
IR (%)	16.6 ± 36.4	31.6 ± 35.5	NS
MR (%)	0% (0/3)	24.7% (19/77)	NS
OPR/ LBDR per ET cycle (%)	21.4% (3/14)	39.2% (58/148)	NS

Values were expressed in means ± SD or in proportions (%)

*Gn* Gonadotropin

^*^Statistically significant (*p* < 0.05)

Our data showed that TPR (21.4% vs 55.4%; *p* = 0.015) and CPR (21.4% vs 52.0%; *p* = 0.029) were significantly lower in the EMT < 8 mm group compared to the ≥8 mm group (Table 2). Excluding the blastocyst transfer cycles, TPR (23.1% vs 55.7%; *p* = 0.025) and CPR (23.1% vs 51.9%; *p* = 0.047) were nevertheless significantly lower in the EMT < 8 mm group for ETs consisted of Day-2/ -3 embryos alone (Table 3). For all that, we did not observe any statistically significant associations between the two groups in terms of BPR, MR and OPR/ LBDR and IR in either all or Day-2 / -3 ETs alone.

**Table 3:** Pregnancy outcomes for Day-2/ -3 ET (excluding blastocyst transfer cycles)

	< 8 mm	≥8 mm	*p*
No. of ET cycles	13	131	Total *n* = 144
TPR (%)	23.1% (3/13)	55.7% (73/131)	0.025^*^
BPR (%)	0% (0/13)	3.8% (5/131)	NS
CPR (%)	23.1% (3/13)	51.9% (68/131)	0.047^*^
IR (%)	17.9 ± 37.5	32.0 ± 35.7	NS
MR (%)	0% (0/3)	22.1% (15/68)	NS
OPR/ LBDR (%)	23.1% (3/13)	40.5% (53/131)	NS

^*^Statistically significant (*p* < 0.05**)**

Pregnancy outcomes of each 1 mm EMT increment were stratified from EMT < 6 mm till ≥19 mm (Table 4). No pregnancy was seen at the extremes of EMT < 7 mm and ≥ 19 mm.

**Table 4:** Pregnancy outcomes by EMT

EMT (mm)	Cycle (n)	TPR (n, %)	BPR (n, %)	CPR (n, %)	IR (%)	MR (n, %)	OPR/LBDR (n, %)
< 6.0	2	0	0	0	0	NA	0
≥6 to < 7	1	0	0	0	0	NA	0
≥7 to < 8	11	3 (27.3)	0	3 (27.3)	21.2 ± 40.2	0	3 (27.3)
≥8 to < 9	24	14 (58.3)	1 (4.2)	13 (54.2)	24.3 ± 31.4	4 (30.8)	9 (37.5)
≥9 to < 10	33	20 (60.6)	1 (3.0)	19 (57.6)	34.8 ± 34.9	5 (26.3)	14 (42.4)
≥10 to < 11	30	16 (53.3)	1 (3.3)	15 (50.0)	33.8 ± 39.7	4 (26.7)	11 (36.7)
≥11 to < 12	22	10 (45.1)	1 (4.5)	9 (40.9)	31.0 ± 40.9	2 (22.2)	7 (31.8)
≥12 to < 13	16	10 (62.5)	0	10 (62.5)	37.5 ± 34.2	2 (20.0)	8 (50.0)
≥13 to < 14	9	6 (66.7)	0	6 (66.7)	42.4 ± 35.4	1(16.7)	5 (55.6)
≥14 to < 15	4	2 (50.0)	1 (25.0)	2 (50.0)	29.0 ± 34.1	0	2 (50.0)
≥15 to < 16	5	3 (60.0)	1 (20.0)	2 (40.0)	23.2 ± 32.3	1 (50.0)	1 (20.0)
≥16 to < 17	2	0	0	0	0	NA	0
≥17 to < 18	2	1 (50.0)	0	1 (50.0)	16.5 ± 23.3	0	1 (50.0)
≥18 to < 19	0	NA	NA	NA	NA	NA	NA
≥19	1	0	0	0	0	NA	0

*NA* Not Applicable

We further divided the EMT ≥8 mm group into two subgroups, ie. EMT ≥8 to < 14 mm vs EMT ≥ 14 mm for comparison of pregnancy outcomes, and found no statistical significance between the two subgroups for all pregnancy outcomes (*p* > 0.05) (Table 5).

**Table 5:** Subgroup comparisons for pregnancy outcomes

	≥8 to < 14 mm	≥14 mm	*p*
No. of ET cycles	134	14	Total *n* = 148
TPR (%)	56.7% (76/134)	42.9% (6/14)	NS
BPR (%)	3.0% (4/134)	7.1% (1/14)	NS
CPR (%)	53.7% (72/134)	35.7% (5/14)	NS
IR (%)	32.9 ± 36.1	18.9 ± 27.4	NS
MR (%)	25.0% (18/72)	20.0% (1/5)	NS
OPR/ LBDR (%)	40.3% (54/134)	28.6% (4/14)	NS

When looked into some risk factors possibly implicated for defective endometrial receptivity, we observed a trend towards higher prevalences of previous PID (14.3% vs 4.1%; *p* = 0.144) and ectopic pregnancy (14.3% vs 7.4%; *p* = 0.312) in the EMT < 8 mm group; however, no statistical significant association was found (Table 6).

**Table 6:** Risk factors for defective endometrial receptivity

	< 8 mm	≥8 mm	*p*
No. of ET cycles	14	148	Total *n* = 162
Previous PID	2 (14.3%)	6 (4.1%)	NS
Previous ectopic pregnancy	2 (14.3%)	11 (7.4%)	NS
Endometriosis	1 (7.1%)	14 (9.5%)	NS
Previous uterine instrumentation / surgery			
- Previous D&C	0 (0%)	0 (0%)	NS
- Previous myomectomy breaching endometrial cavity	0 (0%)	1 (0.7%)	NS
Previous possible endometritis			
- Previous multiple miscarriages	0 (0%)	0 (0%)	NS
- Previous uterine infection post-miscarriage	0 (0%)	0 (0%)	NS

## Discussion

The functional layer of endometrium starts growing under the hormonal influence of estrogen till it reaches a maximum at the onset of the luteinizing hormone (LH) surge. On sonographic assessment, EMT increases in the follicular phase to reach a maximal thickness on the day of ovulation trigger.

Despite many studies demonstrated a higher probability of pregnancy once the EMT attained a threshold value [7, 19–22], others failed to identify any significant relationship between EMT and PRs in ART patients [23–25].

Due to the relatively simple, noninvasive and reproducible advantages of EMT measurement by transvaginal ultrasound as a tool to assess endometrial receptivity, EMT remains one of the most researched parameters for its clinical significance in ART.

Besides EMT, endometrial echo pattern was another frequently used tool to evaluate endometrial receptivity. Endometrium displaying good texture (triple-line) on the day of hCG administration was considered in favour for successful pregnancy in ART cycles as reported in some studies [3, 21, 26, 27]. We had seen differing practice in our own setting with yet no consensus made as to whether or not to defer a fresh ET in patients with TVU findings of non conformance to the triple-line endometrial pattern on the hCG trigger day. As a measure of standardization and in the interest of exploring the EMT threshold in this study, we had included only patients who had demonstrated the triple-line endometrial pattern in the study period for analysis.

For the purpose of defining a ‘thin’ endometrium, various EMT cut-off values had been previously assessed, with most ranging between 6 and 10 mm [2, 8]. For our study, we used an EMT cut-off value of 8 mm, below which, we defined it as a ‘thin’ endometrium. EMT of 8 mm corresponded to the lower 10th centile EMT in our study population, and this cut-off value had also been evaluated previously in few other studies [8, 28]. Based on our hypothesized threshold value, the findings derived from present study would provide better insight and guide us on our daily ART practice.

In our study, we found that TPR and CPR were improved with a minimum threshold EMT of 8 mm on ovulation trigger day. This trend was observed in some previous studies [8] although the threshold values used were not the same. There seemed to be a relationship between increased EMT and better overall PRs. Findings from our study suggested EMT ≥ 8 mm could be a useful indicator of better endometrial receptivity in our population.

After identifying EMT ≥8 mm for having a better prognosis for TPR and CPR, we further divided the group into subgroups of ≥8 to < 14 mm and ≥ 14 mm in order to elucidate the impact of an arbitrary ‘thick’ endometrium with an upper cut-off value of 14 mm. Like in other previous studies, EMT of 14 mm corresponded to the 90th – 95th centile EMT for our study population. We found in our study that all pregnancy outcomes were comparable between EMT ≥8 to < 14 mm and ≥ 14 mm. In contrary to some previous studies which showed a gradual declining trend in PRs above EMT of 14 mm [10, 12], we did not find any detrimental effect of a so-called ‘thick’ EMT in our study, but rather regard such finding per se to be dispensable.

Our study came to define a ‘thin’ EMT as < 8 mm, and to the best of our knowledge within the Southeast Asian region, this cut-off value was being assessed and noted for the first time to be statistically significant with distinguishing effects on pregnancy outcomes. We postulated for an improved TPR and CPR found at EMT threshold higher than the conventional ‘thin’ EMT of 6-7 mm, there could probably be numerous interlacing factors stemming from genetic, ethnic to environmental in origin that were subjected to regional variation that would influence the fundamental buildup of EMT. We did not conduct analysis for any PGS/ PGD cycle or donor cycle and in fact, patients with these cycles were excluded from our study. The morphological grading for embryos transferred was similar between the EMT < 8 mm and ≥ 8 mm groups in our study. We hence postulated that the higher CPR in EMT ≥ 8 mm did not translate into a higher ongoing PR and LBDR or lower MR, to a certain extent, could be related to individual genetic or chromosomal normalcy of transferred embryos.

This Ministry of Health ART scheme was initiated for infertility couples with no biological living children. Importantly to note, we did not have patients with previous D&C, prior multiple miscarriages or previous uterine infection post-miscarriage. Only one patient had a prior myomectomy breaching endometrial cavity. Intrauterine adhesions could be implicated following endometrial trauma directly or indirectly caused by the above mentioned events. Depending on the severity of insult, endometrium might be rendered less receptive to embryo implantation [29]. 9.3% of our patients had endometriosis. Some studies had advocated decreased pro-implantation biomarkers and increased progesterone resistance culminating in defective endometrial receptivity and decreased implantation rates (IRs) in patients with endometriosis [29]. However, these factors did not seem to have any association with our study EMT threshold.

Prior PID and ectopic (including tubal) pregnancy with or without pelvic surgical management affected 4.9% (*n* = 2 in < 8 mm; *n* = 6 in ≥8 mm group) and 8% (*n* = 2 in < 8 mm; *n* = 11 in ≥8 mm group) respectively, of our study population. PID was a clinical spectrum that included salpingitis and endometritis which was closely associated with infertility. Salpingitis might predispose to the development of tubal pregnancy, tubal infertility and defective endometrial receptivity. Salpingectomy of a diseased tube as pointed out was followed by significant improvement in endometrial receptivity markers as assessed by luminal endometrial epithelial αvβ3 integrin expression, indicating the presence of detrimental effect on endometrium rendered by untreated communicating hydrosalpinges; however, the same study also showed post-treatment endometrial glandular epithelial integrin expression, although improved, did not reach statistical significance, suggesting that complete normalization of endometrium did not take place even after salpingectomy [30] . As we assessed what was inherent to the ‘thin’ endometrium in our study population, we noticed a higher prevalence of prior PID and ectopic pregnancy in the EMT < 8 mm group, nevertheless, no statistical significant association was attained.

Miwa et al., 2009 [31] reported that thin EMT were characterized by high uterine blood flow impedance, impaired glandular epithelial growth, decreased vascular endothelial growth factor (VEGF) expression, and thus the vicious cycle of poor vascular development and ‘thin’ EMT. It was explanatory that such ‘thin’ endometrium might adversely affect on endometrial receptivity thus functionally insufficient to support pregnancy development after implantation. As above mentioned, ‘thin’ endometrium could be attributed to many aetiologies including infection, inflammation and iatrogenic causes resulting in fibrosis and decreased endometrial vascularity [32]; however, in our study consisted of its current sample size, none of these factors was statistically proven to be associated with the development of ‘thin’ endometrium. Yet another explanation to this was that a ‘thin’ EMT could just be a coincidental finding, where it simply depicted individual uterine architecture or the intrinsic properties of endometrium and might not be related to disease processes [32]. As shown in our study, although the IRs were not different between the < 8 mm and ≥ 8 mm group, the TPR and CPR were found to be significantly higher in the ≥8 mm group, reinstating the substantial intrinsic role of the EMT component in promoting and perhaps sustaining embryo-endometrium interaction during implantation and pregnancy development.

The general notion is thus that pregnancy outcomes improve with a thicker EMT, however, every result has to be interpreted with caution. What could further enhance our practice based on our present study would be with an EMT < 8 mm, we get to knowingly consider the options and directions of treatment, which also provides us the basis for patient counselling.

The dilemma would still be whether we should now continue treatment in cycles with ‘thin’ EMT or should we cryopreserve the embryos for subsequent FET cycles for potentially better pregnancy outcomes. However, it was noted in our study the benefits of better TPR and CPR with EMT ≥8 mm did not translate into better ongoing PR and LBDR. This would contend us against the decision of calling straight for a fresh ET cancellation. Having said that, another option worth exploring given by Kumbak B et al. [33] would be unless the number of embryos available for transfer ≥ three, the patient could be counselled and offered the option of total embryo freezing.

Besides, the perceived optimal EMT ≥8 mm could be a threshold guidance in the next cycle treatment for women who failed to conceive with ‘thin’ EMT. Avenues that have been looked into by some studies include intra-uterine granulocyte colony-stimulating factor (IU G-CSF) instillation, extended estrogen support in FET cycles, administration of drugs aimed at increasing endometrial blood flow (eg. pentoxyfilline, tocopherol, vaginal sildenafil, l-arginine and low dose aspirin) [32] and endometrial receptivity array (ERA) test to ensure embryos are transferred to a receptive endometrium [34]. However, a lot of these treatments are still under evaluation in ongoing studies and thus not been validated by robust evidence. Besides, further confirmation of our preliminary data is needed before any drastic change in the therapeutic approach could be suggested. For the time being, the treatment modalities are by and large at the physician’s discretion after taking due consideration of all aspects concerning the patient’s pregnancy prospects.

While triple-line endometrium (but not EMT) was a prerequisite to a fresh ET cycle in our setting, thus the results in this retrospective study; varying practice was seen with a non-triple-line endometrium, hence the inability for a combined analysis of EMT and endometrial echo patterns to be carried out in our study involving fresh completed autologous cycles. The inclusion of both Day 2/3 and Day 5/6 ETs was helpful to expand the number of cycles for the study, however, was acknowledged as a study limitation because the day of transfer was known to affect implantation and pregnancy rates. To address and control for this limitation, we had done a subanalysis for pregnancy outcomes for Day 2/3 ET cycles alone, after excluding all blastocyst transfer cycles.

Despite attempts to encompass for a larger study sample size with a five-year retrospective review and measures taken in ensuring better control of confounding factors by imposing certain exclusion criteria, the main limitations of this study were nevertheless a small sample size and patient population heterogeneity. One of the suggestions for future researches would be to minimize embryo quality variation by conducting well-design prospective studies using gamete-donation cycles or PGS/ PGD cycles with replacement of genetically and chromosomally normal embryos in order to better determine the independent effect of EMT as a parameter of endometrial receptivity. Ideally, there should be studies with larger sample size from single centers in this region to further verify the findings.

## Conclusions

Our current findings suggest that EMT < 8 mm on hCG trigger day could adversely affect TPR and CPR in infertile patients undergoing IVF/ICSI-ET, while EMT ≥14 mm does not add advantage or compromise pregnancy outcomes. The study is founded on completed cycles which each has demonstrated triple-line endometrial pattern on the hCG trigger day with fresh autologous ET consisted of high-quality morphological gradings. However, our findings are still preliminary to suggest decision for ET transfer, cycle cancellation or adjunctive therapies. Further studies with a bigger sample size, especially from this region, are required to verify the findings.

## Abbreviations

ART: Assisted reproductive technology
BMI: Body mass index
BPR: Biochemical pregnancy rate
COS: Controlled ovarian stimulation
CPR: Clinical pregnancy rate
D&C: Dilation and curettage
E2: Estradiol
EMT: Endometrial thickness
ERA: Endometrial receptivity array
ET: Embryo transfer
FET: Frozen-thawed embryo transfer
FSH: Follicle-stimulating hormone
Gn: Gonadotropin
GnRH: Gonadotropin-releasing hormone
hCG: Human chorionic gonadotropin
ICSI: Intracytoplasmic sperm injection
IR: Implantation rate
IU G-CSF: Intra-uterine granulocyte colony-stimulating factor
IVF: In vitro fertilization
LBDR: Live birth delivery rate
LH: Luteinizing hormone
MR: Miscarriage rate
NA: Not applicable
NS: Not significant
OPR: Ongoing pregnancy rate
OR: Oocyte retrieval
PGD: Preimplantation genetic diagnosis
PGS: Preimplantation genetic screening
PID: Pelvic inflammatory disease
POR: Poor ovarian response
PRs: Pregnancy rates
SD: Standard deviation
TPR: Total pregnancy rate
TVU: Transvaginal ultrasound
VEGF: Vascular endothelial growth factor
